# Vitamin D Status of Clinical Practice Populations at Higher Latitudes: Analysis and Applications

**DOI:** 10.3390/ijerph6010151

**Published:** 2009-01-07

**Authors:** Stephen J. Genuis, Gerry K. Schwalfenberg, Michelle N. Hiltz, Sharon A. Vaselenak

**Affiliations:** University of Alberta, 2935-66 Street, Edmonton Alberta, Canada; E-Mails: gschwalf@telus.net (G. K. S.); mchiltz@shaw.ca (M. N. H.); svaselenak@interbaun.com (S. A. V.)

**Keywords:** Dietary supplements, epidemiology, hypovitaminosis-D, information science, knowledge translation, nutrition, public health, vitamin D, vitamin D deficiency, vitamin D insufficiency

## Abstract

**Background:**

Inadequate levels of vitamin D (VTD) throughout the life cycle from the fetal stage to adulthood have been correlated with elevated risk for assorted health afflictions. The purpose of this study was to ascertain VTD status and associated determinants in three clinical practice populations living in Edmonton, Alberta, Canada - a locale with latitude of 53°30’N, where sun exposure from October through March is often inadequate to generate sufficient vitamin D.

**Methods:**

To determine VTD status, 1,433 patients from three independent medical offices in Edmonton had levels drawn for 25(OH)D as part of their medical assessment between Jun 2001 and Mar 2007. The relationship between demographic data and lifestyle parameters with VTD status was explored. 25(OH)D levels were categorized as follows: (1) Deficient: <40 nmol/L; (2) Insufficient (moderate to mild): 40 to <80 nmol/L; and (3) Adequate: 80–250 nmol/L. Any cases <25 nmol/L were subcategorized as severely deficient for purposes of further analysis.

**Results:**

240 (16.75% of the total sample) of 1,433 patients were found to be VTD ‘deficient’ of which 48 (3.35% of the overall sample) had levels consistent with severe deficiency. 738 (51.5% of the overall sample) had ‘insufficiency’ (moderate to mild) while only 31.75% had ‘adequate’ 25(OH)D levels. The overall mean for 25(OH) D was 68.3 with SD=28.95. VTD status was significantly linked with demographic and lifestyle parameters including skin tone, fish consumption, milk intake, sun exposure, tanning bed use and nutritional supplementation.

**Conclusion:**

A high prevalence of hypovitaminosis-D was found in three clinical practice populations living in Edmonton. In view of the potential health sequelae associated with widespread VTD inadequacy, strategies to facilitate translation of emerging epidemiological information into clinical intervention need to be considered in order to address this public health issue. A suggested VTD supplemental intake level is presented for consideration.

## 1. Introduction

Recent medical literature is replete with discussion about health benefits of vitamin D (VTD) sufficiency as well as risks for adverse sequelae and disease states associated with inadequate VTD levels (Table 1). Once thought to be primarily a determinant of bone health, it is now recognized that VTD is a requisite hormone involved in myriad physiological processes including immune modulation, genetic regulation, hormonal production, and cellular functioning throughout the life cycle. While ongoing research continues to explore the short and long-term sequelae of prenatal and pediatric VTD deficiency, several clinical studies in adults have linked hypovitaminosis-D with increased risk for various conditions including osteoporosis, assorted cancers, cardiovascular disease, diabetes and others.

Several studies in the literature suggest widespread VTD inadequacy [1], particularly in higher latitudes where ultraviolet B (UVB) sunlight intensity is too weak for extended periods to induce sufficient VTD skin synthesis for many people. Despite the availability of this information, however, the potential lag or latent period between nutrient inadequacy and adverse health outcomes [2], as well as general inattention to clinical nutrition in medical education [3], has contributed to a low index of suspicion in clinical practice about hypovitaminosis-D as an etiological determinant of ill-health. This study was designed to determine the VTD status among three clinical practice populations in Edmonton, a locale in western Canada with latitude of 53°30’N – about the same latitude as Hamburg, Manchester, Liverpool, Dublin, Warsaw, Moscow and Leningrad – where sun exposure is often inadequate to generate sufficient vitamin D from October through March [4].

## 2. Methodology

Three practicing physicians in Edmonton, Alberta, Canada with interest in clinical nutrition were recruited to assess VTD status in their patients. For a period of over five years between June 2001 and March 2007, a generalist Obstetrician/Gynecologist (MDA) collected 25(OH)D (the main circulating metabolite of VTD) levels on all new patients referred for obstetrical or gynecological care. During this same period, a primary care generalist (MDB) ordered 25(OH)D levels if VTD status was clinically deemed to be a potential determinant of the patient’s health problem. The third physician, a part-time family doctor (MDC), collected 25(OH)D levels on consecutive patients presenting for annual check-ups. MDB and MDC have general primary care practices with no decided focus on a specific patient or illness demographic. High-pressure liquid chromatography, mass spectrometry performed at a single core laboratory was used to determine 25(OH)D levels.

Demographic data including patient age, sex, skin tone, body mass index (BMI), gestational status, and date of collection as well as lifestyle parameters including liquid milk consumption, sun exposure, use of tanning salons, medication use, vitamin D and fish oil supplementation, and levels of dietary fish intake were recorded. Skin tone categorization was derived from discussion reported in the medical literature relating to significant differences in VTD status as a function of skin tone [39–44]; First Nations skin tone was classified as an independent category as recent literature suggests that First Nations individuals have a high incidence of VTD deficiency [45–48].

Cochran-Mantel-Haenszel’s row mean score statistic was used to evaluate the relationship between 25(OH)D levels and the demographic/lifestyle parameters. Significance levels were adjusted for the following list of potential confounders: physician, use of VTD and fish oil supplements, recent sun exposure, tanning bed use, and season. VTD status was categorized as follows: (1) VTD ‘deficient’: <40 nmol/L, (2) ‘insufficient’ (moderate to mild): 40 to <80 nmol/L, and (3) ‘adequate’: 80–250 nmol/L. As a subcategory, any cases <25 nmol/L were considered severely deficient – a level repeatedly associated with the development of rickets in children from various jurisdictions [11, 49–51]. The literature has not yet achieved consensus on a 25(OH)D level that maximizes multiple health outcomes as the roles of VTD in myriad physiological processes continue to be uncovered. Although variation exists in the definition of VTD ‘deficiency’ and ‘insufficiency’, levels were chosen to be consistent with other research studies to facilitate epidemiological comparison [52–54].

Fisher’s exact test was used to investigate the relationship between severe deficiency with demographic and lifestyle characteristics. Statistical significance was defined as a p<0.05 for all analyses. All statistical analyses were performed using SAS^®^ Version 9.1 (SAS Institute Inc., Cary, NC). Ethics approval was received from the Health Research Ethics Board at the University of Alberta. Each patient signed a written consent to participate in the study.

## 3. Results

Although variation was found between each group, more than 60% of participants within each clinical practice demonstrated hypovitaminosis-D (Table 2) with 77% of MDA’s patients recording inadequate 25(OH)D levels. As three independent groups, the patients of doctors A, B, and C displayed results that were significantly different from one another - with mean VTD levels in the patients of MDA<MDB (p<0.0001); MDB<MDC (p=0.0007) and MDA<MDC (p=0.0046). MDB had older patients than MDA and MDC (both adjusted p<0.001), and MDC’s patients were older than MDA’s (adjusted p=0.0376). A higher proportion of MDB’s patients used fish oil compared to patients in both other clinical practices (both adjusted p<0.0001).

VTD status according to demographic characteristics of study participants is summarized in Table 3. Darker skin with higher melanin blocks UV penetration and retards VTD synthesis. VTD status correlated significantly with skin tone - First Nations and dark-skinned individuals had particularly low 25(OH)D levels. Consistent with other reports in the literature, VTD status correlated with season - levels in summer and fall were higher than those in spring and winter although not significantly when adjusted for potential confounders.

Lifestyle characteristics as they relate to 25(OH)D level are presented in Table 4 and outcomes are consistent with findings in the literature. Fish ingestion, VTD supplementation, fish oil use, regular consumption of VTD fortified milk, tanning bed use, and level of sun exposure were strongly correlated with 25(OH)D results. With milk consumption, however, preferred levels were realized only with ingestion of more than two glasses per day (both adjusted p<0.001). Supplemental VTD intake of up to 400 IU (often the current recommended intake) had a modest effect on 25(OH)D values with major gains in VTD sufficiency achieved only when supplemental ingestion exceeded 400 IU per day (all adjusted p<0.0001). Weekly fish consumers had higher 25(OH)D levels than fish abstainers (adjusted p=0.0021) and periodic or regular use of tanning beds also resulted in higher VTD values (both adjusted p<0.0001). As expected, 25(OH)D values rose with increasing sun exposure (all adjusted p<0.0001). Participants not using VTD or fish oil supplementation had lower mean 25(OH)D levels while those using tanning beds and high sun exposure had superior VTD values. (Table 5)

A small percentage of patients (48 of 1433 or 3.35%) exhibited severe VTD deficiency - i.e. 25(OH)D values <25 nmol/L. (Table 6) Dark-skinned and First Nations individuals were most at risk while participants using supplemental fish oil or VTD had negligible rates of severe deficiency. Furthermore, milk drinkers were significantly less likely to be severely deficient than non-milk drinkers (p=0.0001) confirming food fortification with VTD as a potent public health measure. Only 2 cases (<1%) of severe deficiency occurred among those with regular sun exposure compared to the 46 (5%) with minimal sun exposure (p<0.0001). No significant relationship was found between severe VTD deficiency and season, doctor affiliation, or fish consumption. Although no significant relationship between BMI and severe deficiency was found, 12% of underweight individuals were severely deficient compared to 2–4% in other BMI categories.

## 4. Discussion

The findings of this study are consistent with results from other VTD research suggesting the reality of a hypovitaminosis-D pandemic in some population groups residing at higher latitudes. Although a limitation of this research is selection bias in the overall sample – as all three physicians have a particular interest in clinical nutrition with a large percentage of MDB’s sample, for example, already using fish oil supplements – the likely reality is that VTD levels in the general population may be even lower than those expressed in the overall sample and may more closely approximate MDA’s sample of newly referred patients. As the sample in each clinical practice is not randomized and represents a sample of convenience, however, it is not possible to conclude from the data that VTD levels in this study are representative of the general population. Within each practice and the overall sample, nonetheless, it is relevant to consider associations between various demographic and lifestyle factors with VTD status.

Recognized reasons for inadequate VTD status include a) insufficient skin synthesis [1]; b) inadequate ingestion; and c) impaired renal synthesis of 1,25-dihydroxyvitamin D resulting from accumulated toxicants such as lead, aluminum and cadmium [55]. It is unknown to what degree toxicant accretion in selected populations such as Aboriginal groups with specific habitat location, lifestyle and dietary practices contributes to VTD related pathology, but recent Centers for Disease Control findings of widespread toxicant stockpiling including heavy metals [56] suggest that further exploration of this issue may be indicated.

The results of this research study are consistent with numerous other studies in that selected lifestyle and demographic characteristics such as supplementation, skin tone, diet, and sun exposure have a profound impact on VTD status; these findings are not an epiphany. For example, various other studies have reported high rates of VTD deficiency in First Nations communities [44–48], where rickets and osteomalacia appear to be rising [57]. To summarize the findings from this study in conjunction with numerous reports and reviews in the medical literature: i) there is a widespread problem of inadequate VTD status in certain population groups particularly those residing at higher latitudes; ii) there has been a clear association between specific demographic factors and VTD status; and iii) innumerable studies confirm the benefits of VTD adequacy in individuals and population groups. Despite the plethora of studies and generally consistent outcomes, however, the widespread problem of hypovitaminosis-D persists. The focus of this discussion, therefore, will be on considerations and strategies related to the desired effect of this study - the application of these results to clinical practice and public health.

### Application of Vitamin D Research to Clinical Practice and Public Health

As well as the existence of widespread VTD inadequacy and ample understanding of causative determinants, emerging research confirms two important observations related to VTD epidemiology. First, there is a plethora of evidence linking adverse health outcomes to hypovitaminosis-D, a particular problem in higher latitudes. Most people produce VTD in response to UVB exposure from the sun - as ozone blocks UVB emission, the rays become progressively less intense at higher latitudes where sunlight has an increasing angle of penetration and travels greater distances through the earth’s atmosphere. The net result is diminished VTD production at higher latitudes, particularly during winter months. Michael Holick, a pioneer in the unfolding VTD story, noted in a recent review paper that living at higher latitudes is associated with an increased risk for developing type 1 diabetes, multiple sclerosis, Crohn’s disease, hypertension, cardiovascular disease, Hodgkin’s lymphoma, and myriad cancers including colon, pancreas, prostate, ovarian, and breast [1] – residents of higher latitudes are also more likely to die from these cancers [1].

Secondly, there is general acknowledgment that simple and low-cost measures have the potential to address the problem of VTD inadequacy. VTD can be obtained from: a) food sources such as fatty fish and fortified foods; b) skin exposure to UVB light from sun or artificial light sources; and c) VTD supplementation. For those unable to obtain adequate sunlight and/or VTD replete or fortified foods, VTD is readily accessible through low-cost supplementation. Regardless of ample epidemiological data confirming hypovitaminosis-D in many population groups, however, there remains a profound lethargy in moving from data dialogue to actual implementation of clinical measures to address this serious public health problem. Despite abundant evidence and available solutions, primary care practitioners are not yet at a place where VTD assessment and intervention is the standard of care. What accounts for this lethargy and how can medical progress be facilitated?

#### a) Professional Lethargy

Knowledge translation is a description of the process whereby evidence from emerging research is adopted into clinical practice [58]. The stark reality in medicine is that, no matter the evidence, there is sometimes serious difficulty translating knowledge from emerging research into day-to-day clinical implementation. [59, 60] Medical history is replete with examples where sluggish dissemination and adoption of innovation has extracted a heavy toll. It was not until his contemporaries died and a new generation of physicians emerged that Semmelweis’ hand washing intervention to prevent transmission of organisms and puerperal sepsis was implemented [61]. It took more than 40 years before Lind’s recommendation to eat citrus as the solution to scurvy took hold [59]. More recently, Nobel Prize recipient Barry Marshall, reflecting on the length of time necessary to incorporate research about H. Pylori in ulcer management remarked, “Was gastroenterology a science or a religion? I decided it was the latter [62].”

With understandable reluctance to embrace new fads, the medical community is sometimes slow to adopt scientific findings when research lacks industry funding and the associated marketing prowess. Reasons to account for the lethargy in knowledge translation include: a) there is circa six million medical articles produced annually and more than 10 000 new randomized trials indexed on Medline each year [63] – it is unrealistic for busy clinicians to be sufficiently apprised of all recent information; b) with non-commercial endeavors, impetus and funding for dissemination of new findings may not be available; and c) typical human behavior includes a reticence to change, particularly if novel information threatens the credibility or support of existing beliefs. This tardiness, however, is in contrast to incorporation of preliminary or suggestive findings supported by industry such as was seen with hormone replacement therapy [64] and some other pharmaceuticals including certain COX-2 inhibitors – where recent concerns about lack of adequate study and adverse outcomes suggest that incorporation into clinical practice may, at times, be too hasty [65]. With no major commercial sponsor, the move to implement VTD assessment and management into widespread clinical practice has not been readily embraced.

#### b) Solar Abstinence

Exposure to UVB rays from sunlight has traditionally been the greatest source of VTD for most individuals. Based on the presupposition that sun exposure causes skin cancer, a cultural mindset has evolved over the last few decades that sun exposure is unhealthy and solar abstinence is preferable. With widespread use of sunblock and adherence to pervasive admonitions to ‘cover up’, much of the western world has endeavored to avoid solar rays. Although sunburn is recognized to be injurious, recent research has challenged the fallacy that judicious sun exposure is harmful and suggests that sensible and regular doses of sunlight are necessary for health and well being [66, 67]. In fact, prudent exposure to sunlight results in VTD production and appears to have a protective influence on various cancers [68]. Furthermore, evidence continues to accumulate that other physiological processes including endogenous serotonin production may also be dependent on direct sun exposure [69].

Widespread use of topical sunblock which significantly reduces VTD skin production, and the increasing trend to remain indoors have contributed to the hypovitaminosis-D pandemic. The sun-phobic mentality needs to be revisited and the medical community should be at the forefront of addressing this pervasive cultural construct.

#### c) Educational Deficiency

Within a medical paradigm of avant-garde robotic surgical procedures and futuristic epigenomic medical research, clinical nutrition is often perceived as simplistic medicine and not within the routine realm of cutting-edge medical professionals. With the misguided notion that populations in western countries are receiving all the nutrients they require from diet, expertise in clinical nutrition is not secured in most medical schools [3] and proficiency in clinical nutrition is often relegated to non-physician groups. Increasing research continues to elucidate, however, that common deficiencies in essential nutrients such as VTD and essential fatty acids [70, 71] are contributing to widespread illness and that current recommended intake values of some nutrients including VTD have not incorporated recent research.

A major determinant in the clinical adoption of new information, however, is the compatibility of research findings with existing beliefs and values – people feel most comfortable when new information fits within the framework of what they know. Correspondingly, many are inclined to ignore or dismiss emerging science, no matter how credible, if findings challenge their cerebral paradigm. Citrus fruit to cure scurvy, for example, and living organisms as the cause of puerperal fever were a tough sell in a medical climate captivated with Galen’s theory of inherited ‘humours’ as the etiology of illness [72]. Ironically, Eijkman’s subsequent discovery of nutrient deficiency as the cause of beriberi was initially rejected [73] as it did not fit into a medical paradigm that had swung to embrace Semmelweis’ teaching on living organisms [61], and Pasteur’s ‘germ theory’ [74] as the predominant cause of disease. In our current medical paradigm, widespread VTD deficiency as a major determinant of infirmity also remains a tough sell in a medical culture where the mindset of ‘genetic predestination’ [75] as the source of most illness rules the day and nutritional interventions continue to be perceived as ‘complementary’ or ‘alternative’ medicine.

Just as architects or building engineers need to understand intricate details of building materials to address difficulties within structures, clinicians need to comprehensively understand how to investigate and manage the status of nutrients - the prerequisite materials of human physiology and repair [2]. With the increasing attribution of much illness to nutritional compromise [76] and rising attention to emerging areas such as nutrigenomics and nutritional epigenetic determinants of illness, it is time to revisit science-based education on clinical nutrition at all levels of medical education.

#### d) *Quo Vadis*

As discussed, the emerging literature in the fields of information science and knowledge translation suggest that diffusion of innovation in medicine tends to be slow. As a result, many patients continue to be denied credible and effective interventions while some continue to receive ineffective therapies. For example, recent study suggests that up to 25% of patients receive treatments deemed to be useless or harmful [63], while up to 40–50% fail to receive evidence-based interventions or therapies in keeping with current scientific evidence [63, 77]. In order to facilitate the process of knowledge translation to achieve widespread VTD sufficiency in individuals and population groups, discussion of the process of change is in order.

The interplay and interdependence of various determinants which govern uptake of research evidence into clinical practice have recently been explored [63, 78–80] – essential knowledge that is effectively utilized by industry. Successful knowledge translation and clinical implementation requires three fundamental steps: 1) Evidence confirming need for change; 2) Change placed in context of users; and 3) Facilitation of change implementation [80]. To fulfill these requirements in an effort to diminish morbidity and mortality related to VTD inadequacy, interventions utilizing knowledge translation research are presented for consideration.

Many studies confirm the existence of widespread hypovitaminosis-D and the potential health sequelae [81]; the need for change is evident. To position any proposed clinical innovation within the context of physician activity requires understanding of typical physician behavior. Change in clinical practice patterns is much more a function of source of information rather than content; clinical minds are most effectively impacted by people and relationships, not research and data [78, 79]. Physicians generally change their practice behavior not in direct response to research evidence in journals but in response to what they perceive their colleagues are doing (the standard of care) [78, 79] – often communicated at impressive educational events by ostensibly credible experts called ‘opinion leaders’ or ‘thought leaders’. Such presentations are designed to communicate a proposal for change that appears evidence-based, feasible, and attractive to attendees.

It might be effectual to organize similar events, where dissemination of recommended directives to regularly secure VTD sufficiency in patients (not unlike performing regular pap smears in women) is advanced to clinicians. Clear instruction on the ease of laboratory screening, interpretation, and intervention for VTD-related issues would facilitate change implementation by making transition practical and effective. Communicating to health practitioners that no complexity of decision-making is required, that no new skills must be acquired, and that no organizational change is necessary may assist the rapidity of adoption of widespread VTD interventions. Discussion of potential rewarding outcomes for patients associated with VTD sufficiency may further enhance knowledge translation.

From a public health perspective, broad-based education with direct appeal to end users has additional potential to address widespread hypovitaminosis-D. Industry has effectively used direct-to-consumer advertising to achieve commercial success – a technique which has been successful in emerging endeavors not tied to industry. A program entitled “Do Bugs Need Drugs”, for example, was piloted in one Albertan community to diminish misuse of antibiotics. With recognition that children can be bio-vectors of information to families and thus to society at large, university students were employed to teach primary school students about appropriate antimicrobial use. With simple instruction about queries to pose when doctors recommend antibiotics as well as information for parents about management of viral infections, antibiotic use precipitously dropped immediately. Similarly, a public awareness campaign and an educational initiative about VTD sufficiency might engender an expectation of clinical care related to VTD assessment as well as facilitating the widespread acquisition of VTD-related knowledge in society.

By establishing a standard of care, Clinical Practice Guidelines (CPGs) have proven to be one of the most effective tools for facilitating clinical change and potentially improving the quality of care [82] - a phenomenon not missed by industry [83]. Development of credible CPGs which delineate expectations of VTD assessment and management may help establish a standard of VTD care and may be instrumental in facilitating change. Additional public health measures might include a consensus statement on sun exposure, updated VTD intake recommendations, and increasing food fortification. Finally, it would seem prudent that medical regulatory bodies - anointed with the mandate of securing and preserving the public interest - ensure that health professionals under their watch are apprised of important but oft ignored medical interventions.

#### e) Recommended Dosing

Suggested VTD supplemental intake levels are currently being discussed by various groups. Although variation may exist, available evidence suggests that 40 IU of daily VTD supplementation will raise serum 25(OH)D by about 1 nmol/L (range 0.7–2.0 nmol/L) [84–86]. As supplemental VTD intake of 1,000 IU generally increases 25(OH)D levels by about 25 nmol/l, additional daily intake of 2,000 IU VTD for individuals in this study would eliminate deficiency and elevate VTD status to adequate levels in most. (Table 7) With superior pre-existing levels in participants with high sun exposure and tanning bed use (Table 5), caution should be exercised to avoid reaching levels considered potentially toxic (>250 nmol/l). In reality, however, toxicity at daily supplemental doses of 2,000 IU for individuals at high latitudes are unlikely as daily doses of 10,000 IU for up to 5 months have failed to cause toxicity [84,87]. In fact, mean 25(OH)D values observed after 20 weeks of 10,000 IU/d VTD supplementation during winter months for individuals at higher latitudes are in the range of 220 nmol/L [87]. To secure healthy VTD values and to assure avoidance of toxicity, measurement of 25(OH)D levels when indicated is available to help direct clinical care.

#### f) Potential Health and Economic Outcomes

The potential magnitude of outcome resulting from interventions to address the pandemic of low VTD can be realized by considering an important epidemiological concept entitled ‘number needed to treat’ (NNT). The NNT, a calculation discussed in a 1988 publication of the *New England Journal of Medicine* [88], refers to the number of patients who need to be treated with a given intervention in order to prevent one additional bad outcome. The NNT is a valuable measure of the practical usefulness of a health care intervention – a measure which incorporates both the incidence of the adverse condition and the effectiveness of the intervention.

For example, a study was recently undertaken to investigate the efficacy in prevention of coronary and stroke events of the most widely used lipid-lowering agent in the world - atorvastatin (Lipitor) - in patients with hypertension but without previous cardiovascular disease or dyslipidemia [89]. Lipid-lowering therapies are the best-selling medicines in history, used by more than 25 million patients throughout the globe, producing $27.8 billion in sales in 2006 – about half of which are associated with atorvastatin. In this clinical trial, 10,305 hypertensive patients were randomly assigned atorvastatin 10 mg or placebo [89]. The clinical trial ran for 3.3 years and after statistical analysis was completed, it was determined that the NNT was 99.7 – that is, close to 100 patients with this clinical scenario would require ongoing treatment for 3.3 years in order to prevent just one cardiovascular event. The study interpretation concluded that “the reductions in major cardiovascular events with atorvastatin are large [89].”

The impact of VTD therapy, however, may be a great deal more significant - as the benefits of VTD sufficiency, according to recent scientific literature, appear to affect a plethora of common conditions in addition to cardiovascular events. (Table 1) If we consider the high incidence of specific diseases associated, in some cases, with VTD inadequacy (including breast, colon, pancreatic and prostate cancer; cardiovascular events, congestive heart failure, hypertension and fatal stroke; assorted musculoskeletal problems; upper and lower respiratory infections; various autoimmune conditions including multiple sclerosis and rheumatoid arthritis; type 1 and type II diabetes; several obstetrical and gynecological difficulties; etc.), juxtaposed with significant risk reductions for many of these illnesses associated with securing VTD sufficiency (Table 1), the evidence is highly suggestive that the NNT to derive noteworthy benefit is very low. Accordingly, routine VTD assessment and management of all individuals presenting for clinical care would yield positive health outcomes in a high proportion of patients. Appropriate VTD care would also relieve the annual economic burden related to VTD inadequacy, which was estimated at $40–$56 billion in America alone in 2004 [90]. It is hard to envisage another science-based opportunity within contemporary medicine that requires such a simple intervention to yield such a huge reward at such low cost and minimal risk.

## 5. Conclusions

As a consequence of VTD inadequacy, there is a notable gulf between health as it is, and health as it could be for many individuals and population groups. Exposure to UVB rays from sunlight is the single greatest source of VTD for most individuals, yet for periods of the year UVB intensity is too weak at some latitudes to induce sufficient VTD skin synthesis for many people. The findings reported in this paper raise concern that in regions of higher latitudes such as Western Canada where UVB production is impaired for winter months, widespread VTD deficiency may be present. Furthermore, in line with existing research, the study found that individual VTD levels correlate with various determinants including nutritional supplementation, skin tone, sun exposure, use of tanning beds, fish ingestion and medical practice patterns. Underweight individuals as well as participants with First Nations and dark skin tone appear to be at particular risk for severe deficiency.

As VTD is well recognized to have genomic activity and to influence numerous cell processes, VTD inadequacy has been correlated with myriad health problems including various cancers, compromised bone health, all-cause mortality, autoimmune dysfunction, cardiovascular disease, respiratory affliction, as well as obstetrical and gynecological pathology. Furthermore, as hypovitaminosis-D is easily identified and corrected, the medical community has an enormous opportunity as well as an ethical imperative to improve individual and public health by advancing measures - including regular VTD supplementation when required - designed to secure VTD adequacy in individuals and populations. There is a huge gap, however, between what we know works and what is actually done - a phenomenon which poses serious threat to the health and well being of individuals and population groups [77].

History suggests that the transition from best evidence to best practice can be arduous [63]. Many patients receive inappropriate or detrimental care because innovation in clinical medicine often proceeds too slowly, despite abundant evidence. If historical precedent persists, emerging VTD information will not be incorporated into clinical practice for years to come. Using research from the fields of information science and knowledge translation, discussion about impediments to advancement in VTD care is presented for consideration along with tangible recommendations including suggested VTD supplemental intake levels to facilitate medical progress.

## Figures and Tables

**Table 1.** Examples of VTD Status and Clinical Outcomes.

Disorder	Study Design or Intervention	Outcome
**Malignancy**		
Colon cancer	Prospective study of colon cancer risk based on levels of 25(OH)D [5]	75–80% reduction in risk for colon cancer for levels between 67.5–102.5 nmol/L [5]
Prostate cancer	Comparison of men whose 25(OH)D levels were below versus above the median (62.5 nmol/L in winter and 80nmol/L in summer) [6]	Significantly increased risk of aggressive prostate cancer (odds ratio = 2.1) [6]
Pancreatic cancer	Dietary intake of 300–450 IU/d VTD compared to intake <150 IU/d [7]	44% reduction in risk for pancreatic cancer [7]
Breast cancer	Pooled analysis of breast cancer risk based on VTD status [8]	50% risk reduction for breast cancer between groups with high (median = 120 nmol/L) vs. low 25(OH)D levels [8]
Lung cancer	Higher levels of circulating 25(OH)D [9]	Improved survival in early stage lung cancer [9]
All cancer incidence in postmenopausal women	Double-blind, randomized placebo-controlled trial: 1100 IU/d of VTD + 1,500 mg calcium vs. placebo group [10]	77% reduction in risk of cancer in years 2–5 after commencing supplementation [10]
**Musculoskeletal**		
Rickets	25(OH)D levels above 25 nmol/L [11]	Resolution of symptoms and signs [11]
Osteomalacia	800 –2,200 IU/d VTD for up to a year [12]	Resolution of pain symptoms [12]
Osteoporosis	25(OH)D levels above 78 nmol/L to maximize benefit [13]	Improved bone density [13]
Prevention of falls in elderly	Review of RCTs and meta-analyses - VTD dose of 700 IU/d [14]	Significant relationship between VTD supplementation and less risk of falls [14]
Idiopathic back pain	Achieving 25(OH)D levels >80 nmol/L [15]	All deficient patients had pain resolution [15]
Nonspecific chronic musculoskeletal pain	Achieving VTD adequacy [16]	67% of patients had complete resolution of symptoms [16]
Dental - tooth loss in elderly	400–600 IU VTD/d and calcium 1,000 mg/d [17]	50% improvement in tooth retention [17]
**Autoimmune**		
Multiple sclerosis	Supplemental VTD of at least 400 IU/d [18]	41% risk reduction for developing MS [18]
Rheumatoid arthritis (RA)	Intake of VTD each day [19]	Greater VTD intake was inversely associated with risk of developing RA [19]
Type I diabetes	VTD supplementation for infants of 2000 IU/d [20]	78% risk reduction for developing Type I diabetes [20]
**Cardiovascular**				
Fatal Stroke (FS)	Correlated 25(OH)D levels with risk for fatal stroke [22]	Highly significant correlation between low 25(OH)D level and risk for FS [22]
Hypertension	Correlated 25(OH)D levels with risk for hypertension [23]	Plasma 25(OH)D levels were inversely associated with risk of incident hypertension [23]
Cardiovascular events	25(OH)D levels <37.5 nmol/L versus those >37.5 nmol/L [24]	Significantly increased risk of incident cardiovascular events (OR = 1.62) [24]
Peripheral Arterial Disease (PAD)	Correlated 25(OH)D levels with risk for PAD [25]	Low 25(OH)D levels are associated with a higher prevalence of PAD. The prevalence ratio for the lowest quartile compared to the highest was 1.80 [25]
**Obstetric/Gynecologic**		
Gestational diabetes	Levels restored to > 80 nmol/L [26]	Marked improvement of insulin sensitivity and insulin production [26]
Birth weight	Additional gestational VTD intake [27]	Birth weight increased by 11g for each microgram of VTD given [27]
Polycystic ovary disease	VTD repletion with additional calcium [28]	Normalized menses >50% of patients [28]
Premenstrual syndrome	Intake of VTD at ~706 IU per day vs. low intake of VTD at ~112 IU/d [29]	40% reduction in symptoms [29]
Pre-eclampsia (PE)	Correlated 25(OH)D levels with risk of PE [30]	Gestational 25(OH)D levels were inversely associated with risk of PE [30]
**Respiratory**		
Upper respiratory infections	600–700 IU given as cod liver oil [31] (also given selenium and omega 3 fatty acids)	50% reduction in incidence of new upper respiratory infections [31]
Lower respiratory infections	Children with <25nmol/L [32]	11 times more likely to experience infection [32]
Cystic Fibrosis (CF)	VTD added to CF bronchial epithelial cells [33]	Induction of cathelicidin production - increased antimicrobial activity against selected pathogens [33]
Seasonal Influenza	Increased levels of 25(OH)VTD [34]	Up-regulates endogenous antibiotics of innate immunity [34]
**Other**		
All cause mortality	Outcomes of grouped patients with 25(OH)D levels < 42 versus those > 59 over 7.7 year period [35]	All cause mortality 2.08 times higher in lower 25(OH)D groups [35]
Aging and age-related disease	VTD measurement correlated with leukocytic telomere length (a predictor for age-related disease) [36]	Higher VTD levels are associated with markers for diminished aging and age-related diseases [36]
Psoriasis	VTD applied as topical cream [37]	Plaque thickness and redness markedly improved [37]
Type II diabetes	Raising level from 25 to 75 nmol/L [38]	Lower 25(OH)D significantly associated with higher risk of insulin resistance, metabolic syndrome and impaired beta cell function [38]

**Table 2.** VTD Status by Clinical Practice and Practice Comparison for Patient Age and Supplement Use.

Clinical Practice		25(OH)D Level by Clinical Practice – n (%)			25(OH)D Level (nmol/L)	
		<40 nmol/L	40 to <80 nmol/L	80–250 nmol/L	Mean	SD
MDA	(n=570)	105 (18%)	335 (59%)	130 (23%)	63.02	26.86
MDB	(n=686)	118 (17%)	309 (45%)	259 (38%)	70.76	29.13
MDC	(n=177)	17 (10%)	94 (53%)	66 (37%)	76.02	31.76
Overall	(n=1433)	240 (16.75%)	738 (51.5%)	455 (31.75%)	68.33	28.95
**Clinical Practice**		**Age Distribution by Clinical Practice – n (%)**				**Adjusted P-value<0.0001**
		**Pediatrics (<19 years)**	**Young Adults (19 to 30 years)**	**Middle Adults (30 to <60 years)**	**Seniors (60 and older)**	
MDA	(n=570)	30 (5%)	99 (17%)	369 (65%)	72 (13%)	
MDB	(n=686)	43 (6%)	56 (8%)	280 (41%)	307 (45%)	
MDC	(n=177)	14 (8%)	17 (10%)	105 (59%)	41 (23%)	
**Clinical Practice**		**Fish Oil Supplement Use by Clinical Practice – n (%)**				**Adjusted P-value<0.0001**
		**No**		**Yes**		
MDA	(n=570)	483 (85%)		87 (15%)		
MDB	(n=680)	449 (66%)		231 (34%)		
MDC	(n=161)	142 (88%)		19 (12%)		
**Clinical Practice**		**VTD Supplement Use by Clinical Practice – n (%)**			**Missing Data**	**Adjusted P-value 0.9926**
		**None**	**50–400 IU**	**>400 IU**		
MDA	(n=570)	272 (48%)	228 (40%)	70 (12%)	0	
MDB	(n=680)	374 (55%)	176 (26%)	130 (19%)	6 (<1%)	
MDC	(n=161)	68 (42%)	83 (52%)	10 (6%)	16 (9%)	

**Table 3.** Vitamin D Status by Demographic Characteristic.

Characteristic	25(OH)D Level – n (%)			Adjusted P-value
	<40 nmol/L	40 to <80 nmol/L	80–250 nmol/L	
Age				0.1996
Pediatric (<19 years)	24 (28%)	45 (52%)	18 (21%)	
Young Adult (19 to < 30 years)	35 (20%)	91 (53%)	46 (27%)	
Middle Adult (30 to < 60 years)	136 (18%)	414 (55%)	204 (27%)	
Senior (60 and greater years)	45 (11%)	188 (45%)	187 (45%)	
Sex				0.7648
Male	62 (17%)	185 (50%)	123 (33%)	
Female	178 (17%)	553 (52%)	332 (31%)	
Skin Tone				<0.0001
Dark	8 (44%)	6 (33%)	4 (22%)	
Midcolor	43 (23%)	95 (51%)	47 (25%)	
Light	173 (15%)	621 (53%)	385 (33%)	
First Nations	16 (48%)	11 (33%)	6 (18%)	
Pregnant (Females Only)				0.8298
No	159 (16%)	508 (52%)	310 (32%)	
Yes	19 (23%)	44 (53%)	20 (24%)	
BMI (n=704)				0.2959
Underweight (BMI≤18.5)	8 (24%)	17 (52%)	8 (24%)	
Normal (18.5<BMI<25)	51 (16%)	181 (56%)	90 (28%)	
Overweight (25≤BMI<30)	31 (14%)	118 (54%)	68 (31%)	
Obese (BMI≥30)	29 (22%)	78 (59%)	25 (19%)	
Season				0.1447
Spring (March-May)	94 (22%)	220 (51%)	121 (28%)	
Summer (June – August)	30 (10%)	170 (57%)	99 (33%)	
Fall (Sept – November)	38 (12%)	163 (50%)	123 (38%)	
Winter (December – February)	78 (21%)	185 (50%)	110 (29%)	

Note: some of the % do not add up to 100% due to rounding.

**Table 4.** Vitamin D Status by Lifestyle Characteristic.

Characteristic	25(OH)D Level – n (%)			Adjusted P-value
	<40 nmol/L	40 to <80 nmol/L	80–250 nmol/L	
Glasses of Milk per day				<0.0001
None	151 (21%)	353 (50%)	209 (29%)	
1–2	76 (15%)	273 (55%)	143 (29%)	
>2	13 (6%)	107 (52%)	86 (42%)	
Fish Servings per week				0.0159
0	146 (20%)	400 (54%)	196 (26%)	
1	67 (14%)	235 (51%)	163 (35%)	
>1	26 (13%)	98 (48%)	79 (39%)	
Fish Oil Supplement				<0.0001
No	219 (20%)	588 (55%)	267 (25%)	
Yes	21 (6%)	145 (43%)	171 (51%)	
Vitamin D Supplement				<0.0001
None	204 (29%)	394 (55%)	116 (16%)	
50–400 IU	30 (6%)	284 (58%)	173 (36%)	
>400 IU	6 (3%)	55 (26%)	149 (71%)	
Recent Sun Exposure				<0.0001
Minimal	201 (23%)	454 (52%)	223 (25%)	
Moderate	33 (9%)	197 (54%)	132 (36%)	
Lots of Sun	6 (4%)	82 (48%)	83 (49%)	
Tanning Bed Use				<0.0001
None	238 (19%)	675 (53%)	366 (29%)	
Sometimes	2 (2%)	48 (44%)	59 (54%)	
Regular Use (average ≥1/mos)	0 (0%)	10 (43%)	13 (57%)	
Medications				0.8423
None	103 (18%)	306 (54%)	154 (27%)	
Meds not related to VTD	119 (16%)	384 (52%)	239 (32%)	
Meds known to impact VTD	18 (16%)	43 (39%)	49 (45%)	

Note: some of the % do not add up to 100% due to rounding.

**Table 5.** Vitamin D Status by Significant Lifestyle Characteristics.

Category	25(OH)D Level – n (%)			25(OH)D Level (nmol/L)	
	<40 nmol/L	40 to <80 nmol/L	80–250 nmol/L	Mean	SD
No VTD or Fish Oil Supplementation (n=605)	188 (31%)	335 (55%)	82 (14%)	54.60	25.15
No VTD or Fish Oil Supplementation and No Servings of Fish (n=361)	118 (33%)	195 (54%)	48 (13%)	53.74	24.41
No VTD or Fish Oil Supplementation, No Fish Servings and Minimal Sun Exposure (n=231)	98 (42%)	123 (53%)	10 (4%)	46.60	19.39
Tanning Bed Use (n=132)	2 (1.5%)	58 (44%)	72 (54.5%)	86.32	31.59
Tanning Bed Use and Lots of Sunlight (n=26)	0 (0%)	10 (38%)	16 (62%)	102.27	42.12

**Table 6.** Severe Vitamin D Deficiency by Demographic and Lifestyle Characteristic.

Characteristic	25(OH)D Level – n (%)		P-value
	<25 nmol/L	≥ 25 nmol/L	
Age			0.1656
Pediatric (<19 years)	4 (5%)	83 (95%)	
Young Adult (19–29 years)	8 (5%)	164 (95%)	
Middle Adult (30–59 years)	28 (4%)	726 (96%)	
Senior (60+ years)	8 (2%)	412 (98%)	
Sex			0.4022
Male	15 (4%)	355 (96%)	
Female	33 (3%)	1030 (97%)	
Skin Tone			<0.0001
Dark	4 (22%)	14 (78%)	
Midcolor	13 (7%)	172 (93%)	
Light	25 (2%)	1154 (98%)	
First Nations	6 (18%)	27 (82%)	
Pregnant (Females Only)			0.3198
No	29 (3%)	948 (97%)	
Yes	4 (5%)	79 (95%)	
BMI (n=704)			0.0730
Underweight	4 (12%)	29 (88%)	
Normal	9 (3%)	313 (97%)	
Overweight	8 (4%)	209 (96%)	
Obese	3 (2%)	129 (98%)	
Fish Oil Supplement			0.0004
No	46 (4%)	1028 (96%)	
Yes	2 (1%)	335 (99%)	
Vitamin D Supplement			<0.0001
None	45 (6%)	669 (94%)	
50–400 IU	2 (0.4%)	485 (99.6%)	
>400 IU	1 (0.5%)	209 (99.5%)	
Glasses of Milk per day			0.0001
None	38 (5%)	675 (95%)	
1–2	9 (2%)	483 (98%)	
>2	1 (0.5%)	205 (99.5%)	
Recent Sun Exposure			<0.0001
Minimal	46 (5%)	832 (95%)	
Moderate	1 (0.3%)	361 (99.7%)	
Lots of Sun	1 (0.6%)	170 (99.4%)	
Overall	48 (3%)	1385 (97%)	

**Table 7.** Projected Vitamin D Status with Daily VTD Supplementation*.

Category	25(OH)D Level – n (%)			25(OH)D Level (nmol/L)		
	<40 nmol/L	40 to <80 nmol/L	80–250 nmol/L	>250 nmol/L	Mean	SD
Current	240 (16.75%)	738 (51.5%)	455 (31.75%)	0 (0%)	68.33	28.95
Current plus 1,000 IU^a^	8 (1%)	477 (33%)	948 (66%)	0 (0%)	93.33	28.95
Current plus 2,000 IU^b^	0 (0%)	100 (7%)	1,332 (93%)	1** (<1%)	118.33	28.95

a Current plus 1,000 IU is the case where every participant takes an additional 1,000 IU/d VTD

b Current plus 2,000 IU is the case where every participant takes an additional 2,000 IU/d VTD

* Projections based on available evidence that 40 IU/d of VTD supplementation will raise serum 25(OH)D by about 1 nmol/L [84–86].

** One individual’s projected levels went above 250 nmol/L with supplementation. This individual had high sun exposure, regular tanning bed usage, and no supplementation.
